# Quick In Vitro Screening of PGPMs for Salt Tolerance and Evaluation of Induced Tolerance to Saline Stress in Tomato Culture

**DOI:** 10.3390/microorganisms13020246

**Published:** 2025-01-23

**Authors:** Lucas Arminjon, François Lefort

**Affiliations:** Plants and Pathogens Group, Research Institute Land Nature Landscape, HEPIA Geneva School of Engineering Architecture and Landscape, HES-SO University of Applied Sciences and Arts Western Switzerland, 150 Route de Presinge, 1254 Jussy, Switzerland

**Keywords:** *Bacillus megaterium*, *Gliomastix murorum*, *Cryptococcus*, plant-growth promoting microorganisms, PGPM, plant growth-promoting rhizobacteria, PGPR, saline stress, salt tolerance, abiotic stress, salinity

## Abstract

Soil salinity, affecting 20–50% of irrigated farmland globally, poses a significant threat to agriculture and food security, worsened by climate change and increasing droughts. Traditional methods for managing saline soils—such as leaching, gypsum addition, and soil excavation—are costly and often unsustainable. An alternative approach using plant growth-promoting microorganisms (PGPMs) offers promise for improving crop productivity in saline conditions. This study tested twenty-three bacterial strains, one yeast, and one fungal strain, isolated from diverse sources including salicornia plants, sandy soils, tomato stems or seeds, tree leaves, stems, and flowers. They were initially submitted to in vitro selection tests to assess their ability to promote plant growth under salt stress. In vitro tests included auxin production, phosphate solubilization, and co-culture of microorganisms and tomato seedlings in salt-supplemented media. The *Bacillus* sp. strain 44 showed the highest auxin production, while *Bacillus megaterium* MJ had the strongest phosphate solubilization ability. *Cryptococcus* sp. STSD 4 and *Gliomastix murorum* (4)10-1(iso1) promoted germination and the growth of tomato seedlings in an in vitro co-culture test performed on a salt-enriched medium. This innovative test proved particularly effective in selecting relevant strains for in planta trials. The microorganisms that performed best in the various in vitro tests were then evaluated in vivo on tomato plants grown in greenhouses. The results showed significant improvements in growth, including increases in fresh and dry biomass and stem size. Among the strains tested, *Gliomastix murorum* (4)10-1(iso1) stood out, delivering an increase in fresh biomass of 94% in comparison to the negative control of the salt modality. These findings highlight the potential of specific PGPM strains to enhance crop resilience and productivity in saline soils, supporting sustainable agricultural practices.

## 1. Introduction

Many soils worldwide are impacted by excessive salinity, which can manifest as saline (where all mineral salts affect conductivity) or sodic (characterized by high exchangeable sodium levels). This excess salinity can arise from both natural processes and anthropogenic activities, particularly agricultural practices. Notable contributing practices include (1) the application of agricultural inputs and fertilizers [1,2]; (2) irrigation with low-quality water [3,4,5]; and (3) specific agricultural methods, such as deep plowing, or cultivation of salt-tolerant plant species [6].

Approximately one billion hectares of soil worldwide—nearly 9% of the Earth’s land area—are affected by excess salinity [7]. This issue affects 20 to 50% of irrigated agricultural lands [7,8,9], equating to about 60 million hectares (roughly the area of France) [6,8]. The consequences are profound, impacting 1.5 billion people and resulting in an estimated annual economic loss of USD 27.3 billion [9]. Soil salinization poses a growing threat to agricultural productivity [1,4] and food security [6], particularly in arid, semi-arid, and coastal regions [5]. This problem is intensified by climate change, which increases the frequency and duration of drought conditions [8]. The resulting rise in poverty and loss of livelihoods for many smallholder farmers underscores the urgency of addressing this issue. Considering the decreasing arable land and concomitant worldwide population increase, the use of infertile land presents a significant opportunity [10].

In saline soils, plant growth is adversely affected in multiple ways: (1) salinity disrupts the balance of the soil solution, hindering mineral assimilation by plants [11,12]; (2) there is a toxic accumulation of ions such as Na^+^ and Cl^−^ in plant tissues, disrupting ionic and osmotic homeostasis [13,14]; (3) water absorption by roots is limited, leading to cellular plasmolysis [15]; (4) the production of reactive oxygen species (ROS) is induced [1]; and (5) the synthesis of various phytohormones [16], including ethylene, can have detrimental effects on plant growth. In tomato cultivation, for instance, salt stress (at 150 mM NaCl) has been shown to reduce both root and shoot growth [17].

Traditional methods for ameliorating saline soils—such as flushing, leaching, and excavation (physical methods) or the addition of gypsum and lime (chemical methods)—have proven ineffective, costly, and unsustainable [6,18]. While the development of salt-tolerant plant varieties shows promise, it is insufficient alone to meet the food production challenges in salinized areas [6,19]. The application of plant growth-promoting microorganisms (PGPMs) presents a complementary strategy to enhance productivity in saline soils [1,20,21,22,23,24].

PGPMs—including arbuscular mycorrhizal fungi (AMF), plant growth-promoting rhizobacteria (PGPR), and yeasts—support plant growth and alleviate stress through several mechanisms: (1) they produce phytohormones (such as indole-3-acetic acid (IAA) abscisic acid (ABA), ethylene (ET), cytokinin (CK), and jasmonic acid (JA)), which play crucial physiological roles in salt-exposed plants [25,26,27,28]; (2) they reduce the absorption of Na^+^ and K^+^ while enhancing the uptake of essential nutrients [29,30]; (3) they produce ACC deaminase, an enzyme that helps degrade ethylene precursors [31,32,33]; (4) they synthesize exopolysaccharides that protect roots and improve mineral absorption [34,35]; (5) they facilitate the assimilation of osmoprotectants, thus reducing osmotic stress [36,37,38]; and (6) they induce the synthesis of antioxidant enzymes, thereby mitigating oxidative stress [39,40,41].

This study had several objectives. Firstly, it aimed to isolate strains from plants grown in saline environments, preferably endophytic strains. Secondly, the aim was to develop effective in vitro selection methods to identify promising candidates for greenhouse trials. Finally, the main objective of this work was to identify high-performance strains capable of promoting plant growth under salt stress conditions. Twenty-three bacterial strains, one yeast strain, and one fungal strain from different sources were studied using three in vitro selection tests: (1) phosphate solubilization, (2) auxin production, and (3) exposure of sterile seedlings to strains on a salt-enriched medium. At the end of these tests, the five most promising candidates were selected for a greenhouse trial in planta on tomato plants to assess their ability to improve resistance to salinity on *Solanum lycopersicum*. Tomato was chosen as a model plant in this study since it is a moderately salt-tolerant species, widely grown worldwide, and a convenient experimental plant since F1 varieties are available.

## 2. Materials and Methods

### 2.1. Isolation and Identification of Microorganisms from Diverse Sources

The microorganisms tested come from two sources: (1) salicornia plants grown on saline soils and (2) other microorganisms isolated from diverse sources. All sources are given in Table 1 for the latter microorganisms.

#### 2.1.1. Isolation of Endophyte Microorganisms from Salicornia

Samples of *Salicornia europaea* stems and roots, cultivated on saline soils in the Algarve region of Portugal, were collected in 2 cm sections. Some sections were rinsed twice with sterile distilled water in a 250 mL Erlenmeyer flask for 1 and then 3 min. Then, 5 fragments of 2 mm were taken from the center of the sections and cultured on different agar media in 90 mm diameter Petri dishes: Tryptone soya agar (TSA; Roth AG, Arlesheim, Switzerland), Luria Miller agar (LBA; Roth AG), and yeast extract agar (YEA, Roth AG).

Other sections were disinfected with a 2.5% sodium hypochlorite solution and 0.01% Tween 20 for 3 min in a 250 mL Erlenmeyer flask. They were then rinsed three times in succession with sterile distilled water for 1, 3, and 5 min. After drying on two layers of sterile filter paper, 5 fragments of 2 mm were removed with a sterile scalpel, avoiding the ends oxidized by disinfection. These fragments were ground with a sterile mortar and pestle, adding 2 mL sterile 1× PBS (Phosphate-buffered saline; Roth AG). The resulting mixture was then serially diluted to 10^−3^ in sterile 15 mL tubes. Each dilution, together with the initial solution, was collected at 100 µL per dish and spread with sterile rakes on 90 mm diameter Petri dishes containing the same media as above.

Plates were sealed with Parafilm^TM^ (Roth AG) and incubated for 120 h at 22 °C in the dark. Fifteen single bacterial strains were isolated in pure culture and subcultured for 7 days using the same procedures as described above. Out of the 15 isolated bacterial strains from salicornia, 3 were selected for in vitro screening tests (see Section 3.1).

#### 2.1.2. DNA Extraction

From pure cultures on agar, liquid cultures were produced in sterile 15 mL tubes (Roth AG) in the following liquid media: LB Broth, TS Broth, and YE Broth (all from Roth AG). These cultures were incubated under horizontal shaking at 120 rotations per minute (rpm) for 24 h at 22 °C and then centrifuged at 8000 rpm for 4 min. After discarding the culture supernatant, a CTAB-based DNA extraction buffer [42] was added to the bacterial pellets. After purification and resuspension in ultrapure water, nucleic acid quantification was performed using a NanoDrop ND-1000 nanospectrophotometer (Thermo Fisher Scientific, Geneva, Switzerland).

#### 2.1.3. Other Microorganisms

Twenty-two other microorganisms, from the Plants and Pathogens laboratory (P&P laboratory) collection, were selected for the trials. These included 20 bacterial strains, a yeast, and a filamentous fungus (see Table 1), with UASWS codes, standing for University of Applied Sciences and Arts, Western Switzerland. DNA extraction from these strains was carried out in the same way, with the exception that the fungus was ground in liquid nitrogen before the extraction buffer was added.

They were isolated during various campaigns before March 2022 and kept for their potential as plant biostimulants, their endophytic properties, or their origins. There were 10 strains of the *Bacillus* genus, 3 of the *Pantoea* genus, 2 of the *Paenibacillus* genus, and single strains of *Kocuria rhizophila*, *Lysinibacillus* sp., *Microbacte-rium* sp., *Pseudomonas* sp., and *Variovorax* sp. A strain of fungus (*Gliomastix murorum*) and a yeast (*Cryptococcus* sp.) were also selected. Finally, 6 of the 25 selected strains were isolated from *Solanum lycopersicum* seeds or roots.

#### 2.1.4. PCR and Sequencing

Polymerase chain reaction (PCR) amplifications were performed in a total reaction volume of 25 μL in a Biometra ^®^ thermocycler (Goettingen, Germany). Target DNA was used at a final concentration of 1 ng/μL in the reaction mixture. PCR reactions were performed with MyTaq™ DNA polymerase (LabGene, Chatel St-Denis, Switzerland). Primers 27F and 1492R were used for bacterial identification [43] and were produced at Microsynth (Balgach, Switzerland). Amplification conditions for 16S rDNA gene amplification are illustrated in Table 2.

PCR products were purified prior to sequencing using the Wizard ^®^ SV Gel and PCR Clean-Up System kit (Promega, Dübendorf, Switzerland) and sent for Sanger sequencing at Microsynth. The resulting sequences were then edited with FinchTV v.1.5.0 (https://digitalworldbiology.com/FinchTV, accessed on 26 December 2024) and registered in the nucleotide database of the National Center for Biotechnology Information (NCBI, Bethesda, MD, USA) under accession numbers MZ397890 to -MZ397904 for bacterial isolates. DNA sequences were compared with sequences in the NCBI nucleotide database using the BLASTn tool (https://blast.ncbi.nlm.nih.gov/Blast.cgi?PROGRAM=blastn&PAGE_TYPE=BlastSearch&LINK_LOC=blasthome, accessed on 16 June 2021) [44]. Fungal DNA sequences were obtained following the same procedure. PCR amplifications were performed with primers ITS 4 and ITS 5 [45]. PCR amplification conditions are shown in Table 3.

### 2.2. In Vitro Screening

In planta tests are usually time-consuming, space-consuming, labor intensive, and do not always allow for uncovering the mechanisms of action involved in growth promotion. We therefore chose to carry out 3 miniaturized characterization and screening in vitro tests, combining plant and microorganism cultivation, to identify the most promising strains for promoting tomato growth under salt stress conditions.

#### 2.2.1. In Vitro Exposure of Seedlings to Salt and Strains

**Figure 1:**
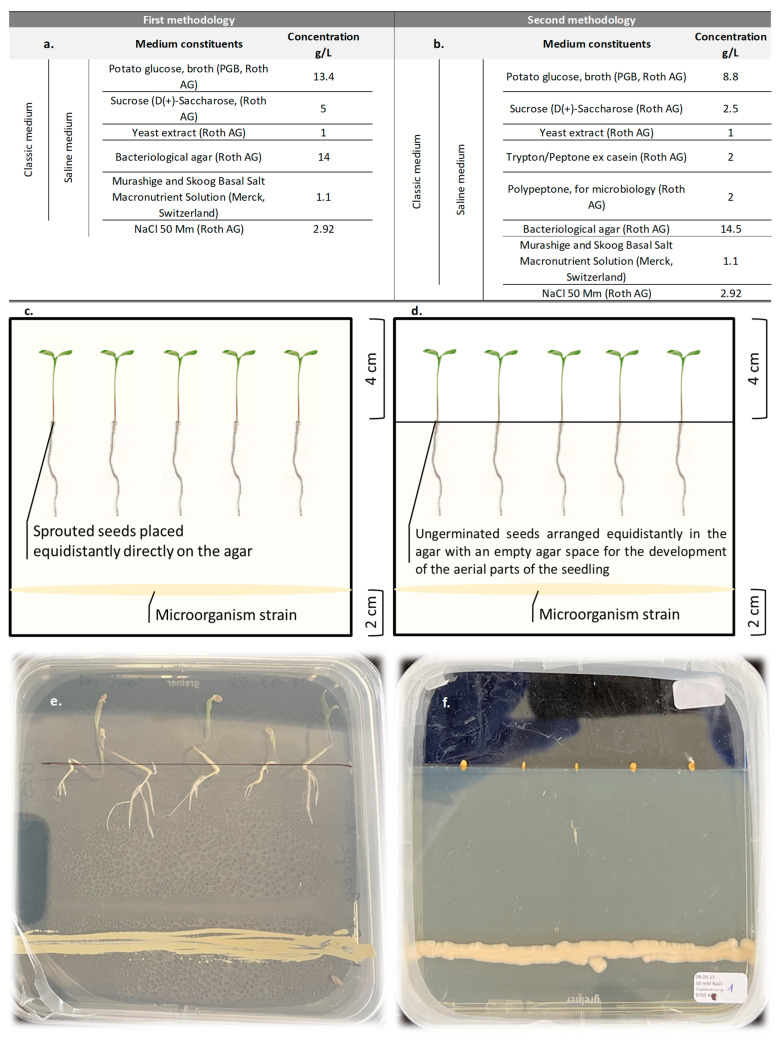
Media composition ((**a**) method 1, (**b**) method 2), experimental set-ups ((**c**) method 1, (**d**) method 2) of the two methods used for in vitro co-cultivation of microorganisms and seedlings. Examples of method 1 with *Bacillus safensis* (**e**) and method 2 with *Cryptococcus* sp. (**f**), 4 days after trial set-up.

This biotest was adapted from Fu et al. [46]. The principle is to bring together a pure culture of a microorganism and tomato seedlings from previously disinfected seeds on the same medium. This in vitro method allows small-scale cultures on a sodium chloride-enriched medium to simulate sodium salt stress for comparison with no-salt controls. If growth promotion mechanisms are not clearly identified using this method, it allows for the clear and quick discrimination of the microorganisms tolerant to salt stress and those that promote seedling growth in the presence of sodium chloride. This experiment was carried out in two stages using two methods (see Figure 1). The 4 most effective strains in all the selection tests combined were used for the second method.

##### Choice of Plant Material and Seed Disinfection

Seeds of *S. lycopersicum* variety Montfavet 63-5 HF1 (Botanic^®^, Gaillard France) were selected. Seeds were disinfected using the following methodology: Seeds were immersed in a 4.5% sodium hypochlorite solution bath with 0.01% Tween 20 added for 10 min, with constant agitation at 140 rpm. The seeds were then drained through a sterile stainless steel strainer and flushed with sterile distilled water for 10 s. Flushing was followed by rinsing three successive times in sterile distilled water baths with constant agitation at 140 rpm for 1 min, 3 min, and 5 min. Seeds were then allowed to dry between 2 sheets of sterile Rotilabo^®^ filter paper (Roth AG), placed in sterile 140 mm diameter plastic Petri dishes (Roth AG) on three layers of sterile Rotilabo^®^ filter paper, and 7 mL sterile distilled water was added for triggering germination. The dishes were then sealed with three layers of Parafilm^TM^ and placed in a germinator (Percival Scientific, Perry, IA, USA) at 26.5 °C and 80% RH: (1) for the first method: 56 h for germination until the appearance of the radicle; (2) for the second method: 48 h for pre-germination.

##### Preparing the Culture Medium

The aim of the medium is to ensure the growth of both the microorganism strain and the plant seedlings. Numerous pre-tests were necessary to develop this medium.

For the first method, the base medium is potato glucose agar (PGA, Roth AG), a commonly used nutrient medium that contains no sodium chloride. Other ingredients included sucrose (D(+)-saccharose, Roth AG), yeast extract (Roth AG), Murashige and Skoog Basal Salt Macronutrient Solution (Merck, Darmstadt, Germany), and bacteriological agar-agar (Roth AG). The final agar concentration was 14 g/L. A salt-enriched version of the same medium was at a final concentration of sodium chloride broth of 50 mM NaCl (Roth AG).

For the second method, a richer medium was elaborated, with a reduced sucrose dose added with Trypton/peptone ex casein (Roth AG) and polypeptone (Roth AG). The final agar concentration was 14.5 g/L. Two versions of this medium were also available with and without salt at the same concentration as above. All media were adjusted to pH 6.5. Precise recipes of these media can be found in Figure 1. After autoclaving, the media were poured into sterile 12 cm × 12 cm square Petri plates (Greiner Bio-One, Roth AG).

##### Experimental Set-Up

For the first method, in each plate, 5 homogeneously germinated seeds were placed on the agar in the upper third of the plate (Figure 1). Each strain is randomly allocated a plate containing the medium without salt and a plate with salt. Each experimental unit, consisting of a 5-seed dish, is repeated twice.

For the second method, the top third of the agar is removed using a sterile dissection scalpel. The 5 seeds are planted in the agar at the interface of the cut. The 4 best strains, identified from method 1, are randomly allocated two plates containing the salt-free medium and two plates with salt. Each experimental unit, consisting of a box of 5 seeds, is repeated twice by modality.

In both cases, the yeast and bacterial strains are spread out in a line two centimeters from the bottom edge of the dish using a sterile loop. The fungus strain is spread in the same position using a sterile scalpel, which is used to scrape propagules from the mother dish. Negative controls are made up of the same device without the microorganisms and are repeated twice.

The work is carried out under sterile conditions in a vertical laminar flow hood MSC 1.2 (Thermo Fisher Scientific). All boxes were sealed with Parafilm^TM^.

##### Growing Conditions and Measurements

The square Petri plates were arranged vertically in a climate chamber (Percival Scientific). Growing conditions were as follows: day: 16 h; T °C: 25 °C; Hr: 60%; light intensity: 70 µmol/s, night: 8 h; T°C: 22 °C; Hr: 60%.

For the first method, the length of the main root and the number of secondary roots were recorded, and photographs were taken during each survey.

For the second method, germination rate, main root length, number of secondary roots, stem length, cotyledon emergence, and number of leaves were recorded. Photographs are taken at each survey too.

In both experiments, the trials were run for 4 weeks, with a reading every week.

#### 2.2.2. Phosphate Solubilization

The strains were evaluated for their ability to solubilize phosphate using a protocol adapted from Nautiyal’s qualitative method [47], using the liquid medium NBRIP-BPB (National Botanical Research Institute’s phosphate-bromophenol blue), enriched with tricalcium phosphate Ca_3_(PO_4_)_2_ and adjusted to a pH of 7.

Selected strains were first grown in 10 mL of their respective liquid media (PGB, LBB, TSA) in 15 mL Rotilabo^®^ tubes at 20 °C with gentle agitation (120 rpm) for 48 h. Then, 100 µL of each culture was inoculated into 30 mL of NBRIP-BPB medium in 50 mL Rotilabo^®^ tubes and incubated at 25 °C under agitation (150 rpm) in the dark for 48 h. Two control tubes, without microorganisms, were prepared and subjected to the same conditions.

After incubation, the tubes were centrifuged at 10,000 rpm for 10 min. For each sample, 150 µL of supernatant was transferred to the wells of a 96-well plate (Roth AG). Optical densities (OD) were measured at 590 nm using a Biotek Absorbance Microplate Reader ELx800™ (Agilent Technologies (Schweiz) AG, Basel, Switzerland).

Results are expressed as a percentage decrease in absorbance compared to control cultures.

$$\%OD_{590nm}\;diminution=1-\frac{OD_{590nm}\;\mathrm{culture}\;\mathrm{supernatant}}{OD_{590nm}\;control}\times 100$$

The average of the values from two replicates is calculated.

This test for qualitative assessment of phosphate solubilization works on the principle of the release of organic acid by the strains in the medium in the presence of tricalcium phosphate, which causes the BPB color indicator to react. The qualitative test correlates well with the analytical results for the determination of phosphates solubilized (quantitative test), as reported by Joly et al. [48].

#### 2.2.3. IAA Production

Indole-3-acetic acid (IAA) production was assayed using the Salkowski reagent according to Matsuda et al. [49]. Using the same 48 h liquid starter cultures used for the phosphate solubilization assay, two 50 mL Rotilabo^®^ tubes per strain, containing 30 mL of their respective media (PGB, TSB, and LBB) supplemented with 200 mg/L L-tryptophan (Roth AG), were inoculated with 100 µL of starter culture.

The tubes were then incubated at 25 °C with gentle rotary agitation (150 rpm) in the dark for 48 h. After incubation, the tubes were centrifuged for 10 min at 10,000 rpm, and 1 mL of supernatant was transferred to a plastic dish. To this supernatant, 1 mL of Salkowski reagent (H_2_SO_4_ (7,9 M); FeCl_3_ (12 g/L)) was added (1:1 mixture) and homogenized. The mixture was incubated for 30 min at room temperature in the dark.

Optical densities (ODs) were then measured at 530 nm using a Biomate 160 UV–visible spectrophotometer (Thermo Fisher Scientific). A standard curve was carried out using PESTANAL^®^ indole-3-acetic acid (Sigma-Aldrich, Darmstadt, Germany) to estimate the quantities of IAA present in the supernatants. This standard range comprised six points of decreasing concentration: 25, 12.5, 6.25, 2.5, 1, and 0.25 mg/L.

This method measures the content of indole compounds produced from L-tryptophan [50,51], which is extrapolated in IAA production. Results are expressed in mg_indol group_/L and are the average of the two replicates.

#### 2.2.4. Selection of Strains of Interest for the I Test

The results of the selection tests were used to choose the 5 strains to be tested in the greenhouse in planta trial. The aim of this trial was to assess the ability of the strains tested to promote the growth of tomato plants under conditions of salt stress, induced by the addition of sodium chloride. The 5 best performing strains in the in vitro tests were selected with the following criteria:

Performance in the seedling in vitro test on salt media (methods 1 and 2) > performance in the seedling in vitro test on non-salted media (methods 1 and 2) = performance in the phosphorus solubilization test = performance in the IAA production test.

In addition to these 5 strains, *Bacillus amyloliquefaciens* BA2 [48] was added as a positive microbial control, as this strain is already marketed as a biostimulant product under the trade name Hélès (Agroline Bioprotect, Aesch, Switzerland).

### 2.3. Test in Planta

The in planta trial took place between 27 April 2022 and 8 June 2022, lasting exactly 6 weeks, in a ‘Venlo’ type greenhouse within a chapel 9.60 m wide and 9 m long (height under gutter: 5.80 m; height under ridge: 6.70 m; roof angle: 22°; 4 mm glass cover). The climatic conditions were as follows: 24 °C during the day, ventilation from 26 °C, 18 °C at night, 60% relative humidity, and no artificial lighting. The following values were measured during the test period: average daily temperature: 22.2 °C; maximum temperature reached: 29.4 °C; minimum temperature reached: 17.3 °C; average relative humidity: 59.8% (maximum: 100% and minimum: 32%); average hourly light intensity: 281.4 W/m^2^ (maximum: 1260 W/m^2^). The microorganisms retained for this experiment in planta are shown below in Section 3.1.

#### 2.3.1. Preparation of Inoculation Solutions

*For yeast and bacteria:* A liquid culture (starter) of each of the 5 strains selected for the test was carried out: incubation time 24 h, at 120 rpm in the dark at 25 °C. From this first culture, 4 Rotilabo^®^ centrifugation tubes (Roth AG) of 50 mL, containing 30 mL of appropriate medium (LBB, TSB, PGB) and strain, were inoculated with 100 µL of seeded liquid culture. Its new tube (multiplication) was incubated for 48 h at 25 °C at 120 rpm in the dark.

*For the filamentous fungal strain*: Four Erlenmeyer flasks with 50 mL of PGB liquid medium were incubated with a piece of mycelium (0.5 × 0.5 cm agar plug). The culture was incubated for 5 days with 160 rpm agitation at 28 °C and was then mixed with a blender to obtain mycelium propagules.

At the end of the incubation period, on the day of inoculation in the greenhouse, the liquid cultures were centrifuged: (1) 4000 rpm for 5 min for the bacteria; (2) 3000 rpm for 5 min for the fungus and yeast strains.

The microbial pellets were re-suspended in 40 mL of PBS (Roth AG) for the yeast and bacterial strains and 100 mL of PBS for the fungal strain.

The viability and concentration of the stock inoculation solutions were monitored using the direct cascade dilution technique and by plating on Petri dishes with agar medium. Cascade dilutions up to a dilution factor of 10^8^ were performed. Daughter solutions with a dilution factor of between 10^3^ and 10^8^ were spread using a sterile rake (Roth AG) in triplicate from 100 µL of daughter solution. Colony-forming units (CFUs) were counted 48 h after spreading (Table 4).

#### 2.3.2. Setting up the Trial

A total of 128 2-liter pots (12 × 12 × 20 cm) were filled with ‘Klassman Substrat 1’ (Eric Schweizer AG, Thun, Switzerland) medium-fertilized substrate (fertilization: 0.8 kg/m^3^ of 14-10-18; pH: 6 ± 0.3). This substrate was previously disinfected (thermal disinfection) with a soil sterilizer Harter Sterilo 1K, (Jarditech, Clarens, Switzerland) during a 2 h cycle. The pots were filled using a homogeneous settling method, i.e., standardized settling by dropping the 10 cm high pot 3 times onto a rigid support. Cups 15 cm in diameter were placed under the pots.

Seeds of *S. lycopersicum* variety Montfavet 63-5 HF1 were sown directly in the pots. Three seeds were placed in the center of the pot at a depth of 6 mm in the substrate. Half the pots (64) were irrigated with a 200 mM sodium chloride solution (11.69 g NaCl/L). The principle was to saturate 50% of the useful water reserve (UR) of the substrate with the salt solution. Brown peat was used as a reference base with a UR of 22%. The other half of the pots were irrigated with clear mains water.

#### 2.3.3. Inoculations

There were 8 different treatments: the 5 selected strains, the positive microbial control (BA2 strain), the positive control fertilized with a commercial fertilizer (Wuxal Universal Fertilizer, Cercle des Agriculteurs—Landi, Satigny, Switzerland), and the negative control (no salt, no microorganisms). For each treatment, 16 pots were allocated (8 with salt, 8 without). The inoculation solutions were brought using a 10 mL Gilson pipette (Gilson (Schweiz) AG, Mettmenstetten, Switzerland) directly onto the substrate at seed level; 2 mL of solution were dispensed into each pot for the bacterial and yeast strains, and 5 mL for the fungal strain. The negative control and the fertilized positive control received 2 mL of sterile PBS in each of their pots. The fertilized positive control received the commercial fertilizer solution at a rate of 300 mL/pot and a dilution at 2 mL concentrated fertilizer/liter of water. Finally, all 128 pots were watered with 10 mL of mains water to help the treatments penetrate.

#### 2.3.4. Randomization

The pots are placed on the cultivation table in a completely random fashion (random draw, Minitab software 21.1.0) [52].

#### 2.3.5. Cultural Operations

Throughout the trial, the crop was irrigated so that the substrate was kept within the same humidity range in each pot by manual weighing of the pots. A single operator was employed for this task. The pots were in a closed circuit thanks to the individual dish placed under each pot. No additional fertilizer was applied.

- Fourteen days after sowing, the crops are thinned to one seedling per pot. By convention, the most vigorous seedling was kept. At the same time, the 64 pots in the ‘salt stress’ condition were irrigated with a 400 mM sodium chloride solution (23.36 g NaCl/L, 200 mL/pot).
- Twenty-three and twenty-nine days after sowing, NeemAzal^®^ (Andermatt AG, Grossdietwil, Switzerland) was applied to control thrips.
- Twenty-eight days after sowing, the crops were staked with a wooden rod.

#### 2.3.6. Measurements

During the trial, (1) the diameter at the collar and (2) the chlorophyll content of the leaves were measured every fortnight. An electronic caliper was used to measure the diameter of the stems at the collar. The side with the largest diameter was selected. The Chlorophyll Content Meter CCM-300 (Opti-sciences, Hudson NH, USA) was used to measure the chlorophyll content of tomato leaves. This device gives a total chlorophyll value expressed in mg/m^2^ of leaf (https://www.optisci.com/ccm-300.html, accessed on 26 December 2024) as described previously [53,54]. The last mature leaf appeared and was selected for measurement. The value retained is the average of three measurements taken on three different leaflets present on the last mature leaf.

At the end of the trial, (1) the ‘Normalized Difference Vegetation Index’ (NDVI) was taken on all 8 plants of each modality. To do this, the 8 plants of each type were grouped together and laid out according to a very precise diagram (spacing of plants every 30 cm in two columns of 4 plants) on a white area, and a measurement was taken at 150 cm from the ground with the GreenSeeker (Trimble Agriculture, Westminster, CO, USA). (2) An overall photo is taken of each modality and compared with the respective control. (3) The fresh mass of the aerial parts was taken and (4) the dry mass after drying (96 h at 45 °C in an oven) was also measured.

### 2.4. Statistics

Statistical analyses were carried out with Minitab on the data collected during the in planta greenhouse trial. The Minitab 22.2.0 software was used.

Fresh weights:

A two-factor analysis of variance (ANOVA) was performed. To comply with the test’s application conditions, the following two modalities were removed (presence of atypical values and high variance): *Halomonas* sp. ESSD20 salt stress and positive control fertilized without salt stress. As mentioned by Cobb [55], ANOVA may be carried out under more flexible conditions; in particular, the equality of standard deviations can tolerate exceeding the threshold of ‘2’, especially when the probability of rejecting the null hypothesis is very high. Groupings illustrating differences between treatments were performed using Tukey’s test with a confidence level of 95%. Confidence intervals for treatments that were statistically significant compared with the negative control were calculated using the Bonferroni test at a 95% confidence level. For information purposes, a Kruskal–Wallis test was used to check whether the fertilized positive control (without salt stress) was different from the negative control in the same group. To objectify the results and identify differences between treatments, multiple comparisons were carried out using the ‘Krus MC’ macro within Minitab.

The *t*-test for two independent samples was used to compare the two populations of negative controls as a function of salt treatment, in terms of the fresh and dry biomass of the above-ground parts harvested.

Stem diameter at the collar (measured 5 weeks after the start of the trial):

- Salt stress group: One-factor ANOVA. Two atypical values were removed from the following modalities to comply with the test application conditions: *Bacillus megaterium* MJ and fertilized positive control. These abnormally high values have been excluded due to the atypical angular shape of the collars concerned. Comparisons with the negative control were made using Dunnett’s test (95% confidence level).
- Group without salt stress: Non-parametric Kruskal–Wallis test.

Dry mass:

- Group with salt stress: One-factor ANOVA. Groupings representing differences between treatments were made using Tukey’s test with a 95% confidence level. Confidence intervals for statistically significant treatments compared with the negative control were calculated using Dunnett’s test (95% confidence level).
- Group without salt stress: Kruskal–Wallis test. Multiple comparisons were carried out using the ‘Krus MC’ macro to identify differences between treatments.

Chlorophyll content (measured 5 weeks after the start of the trial):

- Group with salt stress: Non-parametric Kruskal–Wallis test.
- Group without salt stress: One-factor ANOVA. Groupings illustrating differences between treatments were performed using Tukey’s test (95% confidence level). The confidence interval of the statistically significant treatment compared with the negative control was calculated using Dunnett’s test (95% confidence level).

## 3. Results

### 3.1. Identity of Endophytic Microorganisms Isolated from Salicornia

Table 5 shows all the isolated and identified strains, along with their GenBank accession numbers and UASWS codes in our collection. Following culture and phenotypic sorting of microorganism colonies from glasswort samples, 15 bacterial strains were isolated and identified. Out of these, seven originated from samples that had been disinfected and ground, and eight from root sections rinsed with water (Table 5). The genera *Psychrobacter* and *Halomonas* were the most represented, with five and four strains isolated, respectively. Two strains of *Bacillus* sp. and two of *Marinilactibacillus piezotolerans* were also identified. Finally, *Thioclava* sp. and *Pseudoalteromonas* sp. were also isolated. Out of the 15 strains isolated from glasswort, 3 were selected for in vitro selection tests: *Bacillus* sp. ESSD22, *Halomonas* sp. ESSD20, and *Psychrobacter* sp. ESSD23. *Halomonas* sp. ESSD20 was also retained for the in planta test.

### 3.2. In Vitro Screening

#### 3.2.1. In Vitro Exposure of Seedlings to Salt and Strains

For the first methodology, on saline medium, the longest main roots were found with *Halomonas* sp. ESSD20, with an average length of 32 mm (Table 6). *Bacillus* sp. *ESSD22* came second, closely followed by the negative control (no strain), with mean lengths of 25 mm and 23 mm, respectively. All the other strains had lower average main root lengths than the control, with *Bacillus* sp. E and *Bacillus* sp. BCb1 occupying the last positions, at 14 mm and 13 mm, respectively.

In terms of the number of secondary roots per seedling, seedlings exposed to *Cryptococcus* sp. STSD4 had the highest average number (3). *Paenibacillus* sp. 1.2, *Gliomastix murorum* (4)10-1(iso1) and *Halomonas* sp. ESSD20 induced an average number of secondary roots around 2 (2.4, 2.4, and 2.2, respectively). *Bacillus aryabhattai* 5.3, *Pantoea agglomerans* SB6, *Variovorax* sp. P1C1.4, *Bacillus safensis* B23, *Kocuria rhizophila* B3, *Pantoea* sp. PSb5, *Bacillus* sp. 3.5, and *Pseudomonas* sp. SB10 had seedlings with an average number of secondary roots of between 2 and 1. Seedlings in the negative control had on average only one secondary root. All the other modalities showed on average less than one secondary root or none.

On the salt-free medium, *Bacillus* sp. strain 44, *Pantoea* sp. PSb5, *Bacillus* sp. BCb1, and *Kocuria rhizophila* B3 were the modalities whose seedlings had the longest main roots, with mean lengths greater than or equal to 24 mm. The seedlings of the negative control have main roots with an average length of 19 mm. All the other strains produced seedlings with an average main root length of between 23 mm and 16 mm.

In terms of the number of secondary roots, the strains that most favored their growth were *Bacillus megaterium* MJ, *Bacillus subtilis* 4.4, *Bacillus safensis* B23, and *Bacillus* sp. *BCb*1, with an average number of secondary roots of between 3 and 2 (3, 2.8, 2.8, and 2.3, respectively). Seedlings in the negative control had an average of 1 to 2 secondary roots (1.6). Modalities including *Bacillus* sp. *E*, *Bacillus amyloliquefaciens* BA2, *Pantoea agglomerans SB6*, *Paenibacillus polymyxa* BES12, and *Bacillus amyloliquefaciens* CP showed no secondary roots. All the other strains had an average number of roots per intermediate seedling of between 2 and 0.

**Figure 2:**
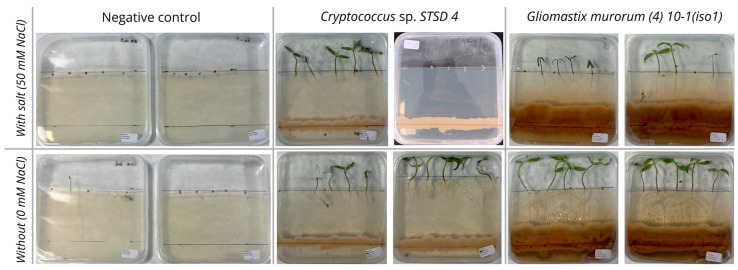
The two best modalities in the seedling in vitro test, with and without sodium chloride (method 2) along with the negative control.

With regard to the second methodology, no germination was observed in the negative control on a salt-enriched medium (Table 7, Figure 2). In the absence of salt, just over half the seeds in the negative control germinated, with a germination rate of 60%. The plantlets obtained had a main root with an average length of 22 mm, with no noticeable branching of secondary roots. The stems were on average 5 mm long, and the appearance of cotyledons was observed in 17% of the germinated seeds.

*Paenibacillus* sp. 1.2, on a salt-enriched medium, induced a germination rate of 20%. The resulting seedlings had small roots at the radicle stage, averaging 2 mm in length, with no secondary roots or stems forming. In the absence of salt, the germination rate is twice as high (40%), and the seedlings developed slightly longer roots (4 mm), still without branching or development of stems.

As with the negative control, no seeds germinated in Petri plates containing *Bacillus megaterium* MJ on a salt medium. On the other hand, 60% of seeds germinated on a salt-free medium. The seedlings obtained had a main root that was shorter on average than that of the control, with an average length of 17 mm and one secondary branch per seedling. The stems were slightly longer, averaging 13 mm in length, with no cotyledons.

*Cryptococcus* sp. STSD4 and *Gliomastix murorum* (4)10-1(iso1) performed the best in this test. Both treatments achieved good germination rates on both saline and non-saline media: 100% and 80% on saline media and 100% and 90% on non-saline media, respectively. Seedlings from these treatments were better developed overall than those from the controls, except for the length of the main roots on a salt-free medium for *Cryptococcus* sp. STSD4 (19 mm compared with 22 mm for the control). However, these roots were more branched, with an average of 7 secondary roots compared with 0 for the control. Significant variability was also observed between the two dishes containing *Cryptococcus* sp. STSD4 on saline medium. In one dish, the seeds germinated without producing well-developed seedlings, in contrast to the other dish where the seedlings developed well, even reaching 100% cotyledon appearance (Figure 2). This event considerably reduced the mean of the parameters measured for this treatment under saline conditions. Hypotheses explaining this variability are described later below in the Discussion Section.

The visual observations in Figure 2 highlight the differences in germination, growth, and interaction depending on the salinity conditions.

#### 3.2.2. Phosphate Solubilization

**Figure 3:**
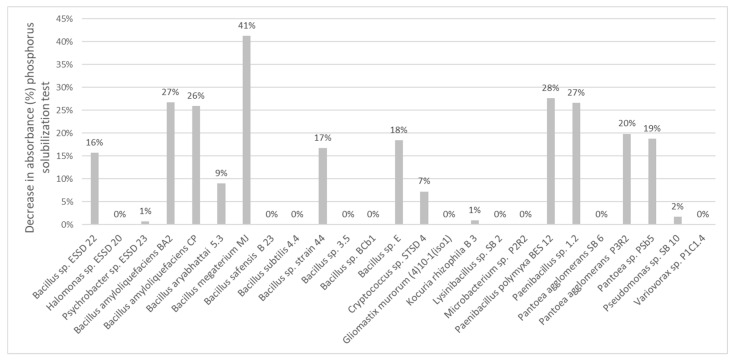
Phosphate solubilization test results expressed as absorbance decrease (in %) compared with the NBRIP control medium.

The sample containing the *Bacillus megaterium* MJ strain showed the greatest decrease in absorbance, reaching 41% (Figure 3). *Paenibacillus polymyxa* BES12, *Bacillus amyloliquefaciens* BA2, *Paenibacillus* sp. 1.2, *Bacillus amyloliquefaciens* CP, and *Pantoea agglomerans* P3R2 also stood out, with a decrease in absorbance of between 30% and 20%.

Samples incubated with *Pantoea* sp. PSb5, *Bacillus* sp. E, *Bacillus* sp. strain 44, and *Bacillus* sp. ESSD22 showed an intermediate absorbance, with a reduction of between 20% and 10%. In contrast, samples containing *Bacillus aryabhattai* 5.3, *Cryptococcus* sp. STSD4, *Pseudomonas* sp. SB10, *Kocuria rhizophila* B3, and *Psychrobacter* sp. ESSD23 showed a slight decrease in absorbance (<10%).

For all other samples, absorbance remained stable, with no significant decrease in bromophenol blue intensity.

#### 3.2.3. IAA Production

The calibration line established from the standard curve obtained a coefficient of correlation (R^2^) of 0.9978.

**Figure 4:**
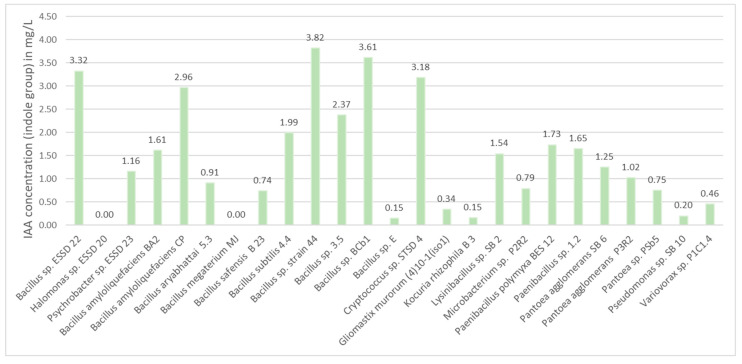
IAA production test (IAA is expressed in indole group equivalents (mg/L)).

The highest producer of indolic compounds was *Bacillus* sp. strain 44, with a concentration of 3.82 mg/L (Figure 4). *Bacillus* sp. BCb1, *Bacillus* sp. ESSD22, and *Cryptococcus* sp. STSD4 followed closely behind, with concentrations above 3 mg/L. *Bacillus amyloliquefaciens* CP and *Bacillus* sp. 3.5 produced slightly lower quantities of indolic compounds, at 2.96 mg/L and 2.37 mg/L, respectively.

In contrast, *Halomonas* sp. ESSD20 and *Bacillus megaterium* MJ did not produce IAA. All the other strains produced intermediate concentrations of indole compounds in the range of 0–2 mg/L.

#### 3.2.4. Selection of Strains of Interest for the in Planta Test

The five best strains (Table 4) were selected based on their performance in the three selection tests:

- *Halomonas* sp. ESSD20 induced the longest main root length (32 mm on average for the five seedlings tested) in the in vitro seedling growth promotion test on a salt-enriched medium (method 1). This strain also performed well in terms of the number of secondary roots in the same test.
- *Bacillus megaterium MJ* showed the best results in the phosphate solubilization test, with a 41% decrease in absorbance.
- *Cryptococcus* sp. STSD4 promoted seed germination and the growth of tomato seedlings in in vitro tests. This strain also ranked highly for IAA production, with a concentration of 3.18 mg/L (fourth among the strains tested).
- *Gliomastix murorum* ((4)10-1 iso 1) also stimulated seed germination and growth of tomato seedlings in the in vitro tests. This strain obtained the best results in method 2 for all parameters, except germination, on a salt-free medium for the average length of the main roots and stem and cotyledon emergence on a salt-enriched medium.
- *Paenibacillus* sp. *1.2* obtained interesting results in the IAA production test (9^e^ position) and the phosphorus solubilization test (3^e^ best strain ex aequo). This strain also showed good results in terms of the number of secondary roots in the test of tomato seedlings in vitro (method 1).

### 3.3. Test in Planta

**Figure 5:**
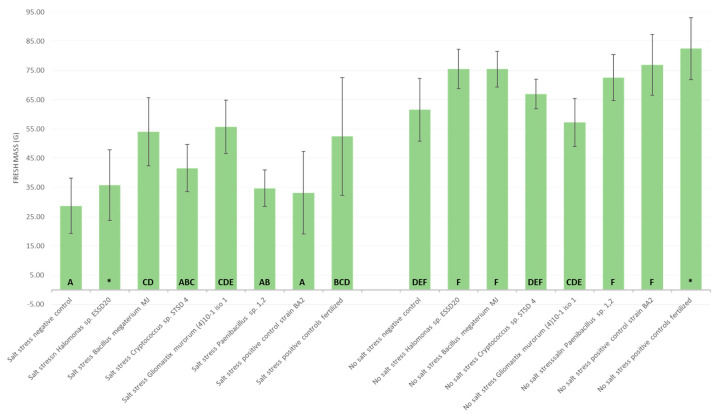
Average fresh mass of the aerial parts. Standard deviations and groupings (Tukey’s method, CI 95%) are shown for each modality. The ‘salt stress’ group is on the left and the ‘no salt stress’ group is on the right (different letters show statistical significance; * mark two modalities not included in Tukey’s).

As shown in Figure 5, after six weeks of cultivation, salt significantly influenced the growth of tomato plants. The fresh biomass of above-ground parts was statistically significantly lower in salt-irrigated plants, with an average fresh mass of 28.68 g per plant, compared with 61.60 g for plants without salt stress (*p* < 0.001, *t*-test for two independent samples). These results are confirmed by the cluster analysis shown in Figure 5 (Tukey’s method). The general breakdown of the data is presented in the form of a graph (Figure A2) in Appendix A.

The same conclusions apply to the dry biomass of above-ground parts: plants exposed to salt have an average dry weight of 3.50 g per plant, compared with 10.90 g for those not exposed (*p* < 0.001, *t*-test for two independent samples).

**Figure 6:**
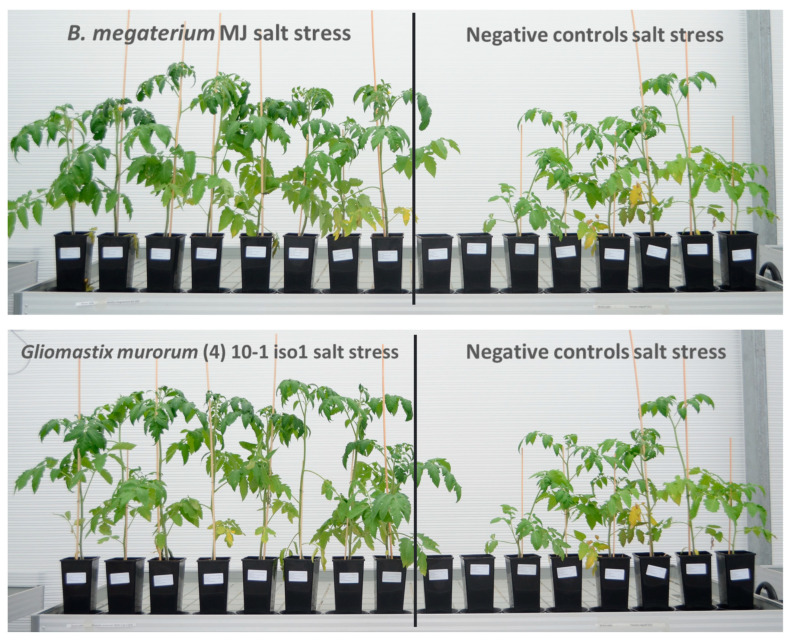
Comparison between the negative controls and the two most effective treatments 6 weeks after starting the experiment.

When comparing the modalities, three treatments stand out from the negative control in the group of plants exposed to sodium chloride (Figure 5). These were *Gliomastix murorum* (4)10-1 iso 1, *Bacillus megaterium* MJ, and the fertilized positive control, with mean fresh weights of 55.74 g (+94% compared to the negative control), 54.01 g, and 52.54 g per plant, respectively. For the best of them, this represents an increase of between +37% and +152% compared with the negative control, thus approaching the performance of the negative control in the non-salt group (Bonferroni simultaneous 95% confidence interval, salt-treated group: *Gliomastix murorum* (4)10-1 iso 1—negative control: [10.62 g; 43.53 g], Table 8). This difference can be seen when all the replicates of the best treatments are lined up against the replicates of the negative control (Figure 6). Although higher than the negative control, the other treatments did not differ in any statistically significant way.

In the group of plants not exposed to salt stress, only the fertilized positive control showed a statistically significant difference, with an average weight per plant of 82.47 g (Kruskal–Wallis multiple comparison chart, Figure A3, in Appendix A). This difference favors the fertilized plants, which produce a higher average fresh mass. Except for *Gliomastix murorum* (4)10-1 iso 1, all the other treatments had a higher average weight per plant than the negative control, although this remains a simple trend. This is particularly the case for the treatment with the positive control strain BA2, which is at the limit of significance (Kruskal–Wallis multiple comparison Figure A3), which has an average weight per plant of 76.93 g.

Finally, two-factor ANOVA for the average fresh mass of the aerial parts of tomato plants showed a significant (*p*-value < 0.001) interaction (Figure A4) between the two factors tested (salt stress and treatment).

For the diameter at the collar, the general breakdown of the data is presented in the form of a graph (Figure A5) in Appendix A. Although the conditions for applying the two-factor ANOVA test were not met, the interaction diagram illustrating the data as a function of the two factors is presented in Appendix A (Figure A6).

In the group of plants irrigated with saline solution, three treatments differed significantly from the negative control. These were *Bacillus megaterium MJ*, *Cryptococcus* sp. STSD 4 and the fertilized positive control, with mean stem neck diameters greater than 0.650 cm, compared with 0.553 cm for the negative control (Table 9).

In the group of plants not exposed to sodium chloride, no statistically significant difference was observed.

For the dry mass, the general breakdown of the data is presented in the form of a graph (Figure A7) in Appendix A. Although the conditions for applying the two-factor ANOVA test were not met, the interaction diagram illustrating the data as a function of the two factors is presented in Appendix A (Figure A8).

In the group irrigated with sodium chloride solution, the same treatments that were statistically different for fresh mass were also statistically different for dry mass. These were *Gliomastix murorum* (4)10-1 iso 1, *Bacillus megaterium* MJ, and the fertilized positive control, with, respectively, mean dry masses of aerial parts of 7.86 g, 6.61 g, and 6.60 g per plant, compared to 3.18 g per plant for the negative control (Table 9).

In the group not exposed to salt, the results differed slightly from those obtained for fresh mass. Two treatments stood out from the negative control: *Halomonas* sp. *ESSD20* and *Paenibacillus* sp. 1.2, with median dry masses of 13.12 g and 13.25 g per plant, respectively, compared with 10.75 g per plant for the negative control (Kruskal–Wallis multiple comparison chart, Figure A9).

For the chlorophyll content, the general breakdown of the data is presented in the form of a graph (Figure A10) in Appendix A. Although the conditions for applying the two-factor ANOVA test were not met, the interaction diagram illustrating the data as a function of the two factors is presented in Appendix A (Figure A11). Few differences were observed between treatments for this parameter, except for the ‘no salt stress’ group, where one treatment stood out from the negative control. This was *Paenibacillus* sp. 1.2, whose plants had an average chlorophyll content in the leaves of 284 mg/m², compared with 241 mg/m^2^ for those in the negative control (Table 9).

The Normalized Difference Vegetation Index (NDVI) generally reflects the results observed for the fresh and dry masses, particularly in the salt-treated group. In fact, the three best treatments mentioned above also had the three best vegetation indices, with values of 0.56 for *G. murorum* (4)10-1 iso 1, 0.57 for *B. megaterium* MJ, and 0.50 for the fertilized positive control, compared with 0.35 for the negative control (Table 9).

In the group not exposed to the saline solution, the results are more nuanced and do not directly reflect those of the fresh and dry masses. The best Normalized Difference Vegetation Index was measured in plants treated with *B. megaterium* MJ, reaching a value of 0.67, compared with 0.47 for the negative control.

In Figure 6, two replicates of the negative control did not even withstand the salt stress applied.

## 4. Discussion

The results presented in this study provide important insights into the interactions between microorganisms and plants under saline conditions and their potential as biological agents promoting plant growth. These results demonstrate significant variation among the different strains, both in terms of their ability to stimulate seed germination and seedling growth and their performance in phosphate solubilization and IAA production tests.

### 4.1. Identification and Selection of Strains

#### Isolation of Endophyte Microorganisms from Salicornia

Six genera are represented among the 15 strains isolated from glasswort tissues. Three of them were selected for the screening tests. Halophilic rhizobacteria of the *Halomonas* genus are known for their plant growth-promoting activities, including under conditions of salt stress [56]. The *Bacillus* genus includes a wide variety of species belonging to the PGPR family. These include *B. velezensis*, some strains of which are well documented for their plant growth-promoting properties, such as strain B25 [48,57]. In addition, several studies have demonstrated the efficacy of certain *Bacillus* strains in stimulating the growth of plants subjected to salt stress [58].

Similarly to *Halomonas*, the *Psychrobacter* genus includes halotolerant bacteria frequently found in the *Salicornia* microbiome. This genus is also known for its PGPR characteristics [59].

### 4.2. In Vitro Screening and Interpretation

The three tests used to select and characterize the microorganisms proved to be particularly effective in identifying potentially interesting candidates for promoting tomato growth under salt stress conditions. Of the five finally selected strains, two showed strong growth-promoting activity in the group of plants exposed to salt stress.

The efficacy and relevance of the phosphate solubilization test (using NBRIP-BPB medium) [47] and the IAA production test using the Salkowski method [49] are well established. These methods, simple to implement, assess two essential mechanisms of PGPRs.

Phosphate solubilization is a key mechanism to improve phosphorus availability, an essential nutrient for plant growth.

On the other hand, other tests may be necessary to assess the microorganisms on other key mechanisms of growth promotion, such as the production of siderophores, the solubilization of other minerals such as potassium, the production of other hormones, or the production of ACC-deaminase. However, these analyses can become time-consuming and costly, particularly when the number of strains to be tested is high.

In this context, the in vitro seedling inoculation test is particularly interesting, as it enables promising strains to be identified in a specific context, such as salt stress in this case, without focusing on a particular mechanism. In fact, it has proved effective in identifying strains of interest that were not identified by the other two in vitro tests. This is particularly true of *G. murorum* (4)10-1(iso1), which showed neither phosphate solubilization activity nor significant IAA production. However, this strain promoted the growth of tomato seedlings in vitro (Table 7 and Figure 2), suggesting the intervention of other mechanisms.

*G. murorum* has been reported as a fungus capable of producing bioactive gibberellins that promote plant growth [60]. Furthermore, the deleterious effects of salt stress on plant germination and growth can be mitigated by the exogenous supply of gibberellins [61]. This test highlights the ability to promote seedling growth in the presence of salt, although the exact mechanisms are not clearly identified. This could include the production of ACC-deaminase [31,32,33], the production of protective exopolysaccharides [34,35], or the reduction in osmotic stress via the absorption of osmoprotectants [36,37,38].

This innovative biotest, which is simple to implement, is based on the method of Fu et al. [46] and adapted to our context. It has proved effective for selecting biostimulant strains in specific situations (such as abiotic stress) and for a particular target plant (such as tomato). In addition, it can be transposed and adapted to study growth promotion through the emission of volatile organic compounds (VOCs) by physically separating the seedlings from the microbial strain. However, improvements are possible to correct certain biases identified during our trials.

Concerning the in vitro test for exposure of seedlings to strains, for the first methodology, the seeds had already germinated when they were placed on the agar. The main root continued to grow on the agar using the seed’s reserves, which is not a good indicator to consider. On the other hand, the appearance of secondary roots only occurred in certain plates, which seems to make it a better indicator.

**Figure A1:**
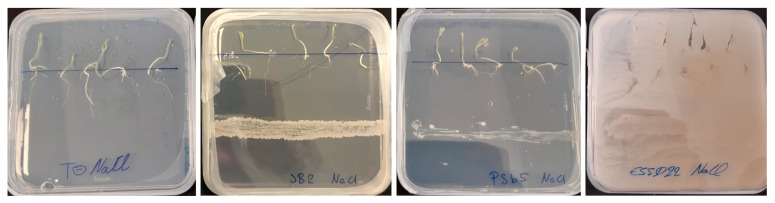
Bias illustration. A bias was identified when inoculating tomato seedlings in vitro using methodology 1. In the pictures taken after 21 d, some bacterial strains showed either weak or excessive growth, colonizing the entire dish on the generic medium used. From left to right, on saline medium: the negative control, a normal-growing strain (*Lysinibacillus* sp. *SB2*), a low-growing strain (*Pantoea* sp. *PSb5*), and a fast-growing strain (*Bacillus* sp. ESSD22). These photos were taken 21 days after the start of the trial.

**Figure A2:**
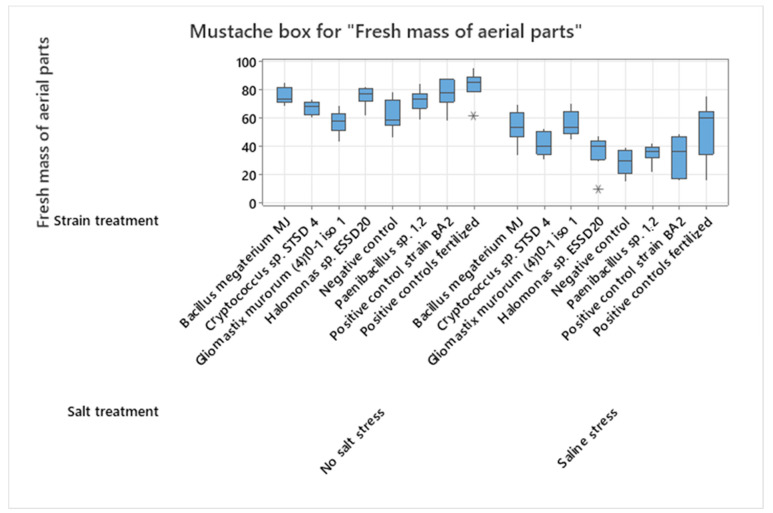
Box-and-whisker plot showing the distribution of fresh biomass data for the above-ground parts of tomato plants according to the different treatments and the two salt-related modalities (* mark the presence of atypical values).

**Figure A3:**
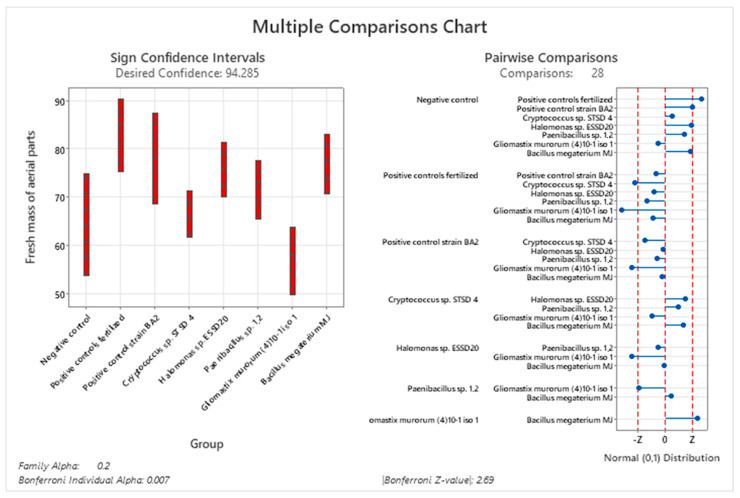
Kruskal–Wallis multiple comparison performed with the ‘Krus MC’ macro. This non-parametric test was used to compare the fresh mass of the aerial parts as a function of the different treatments in the ‘no salt stress’ group of plants. This test compares the ranks of the observations.

**Figure A4:**
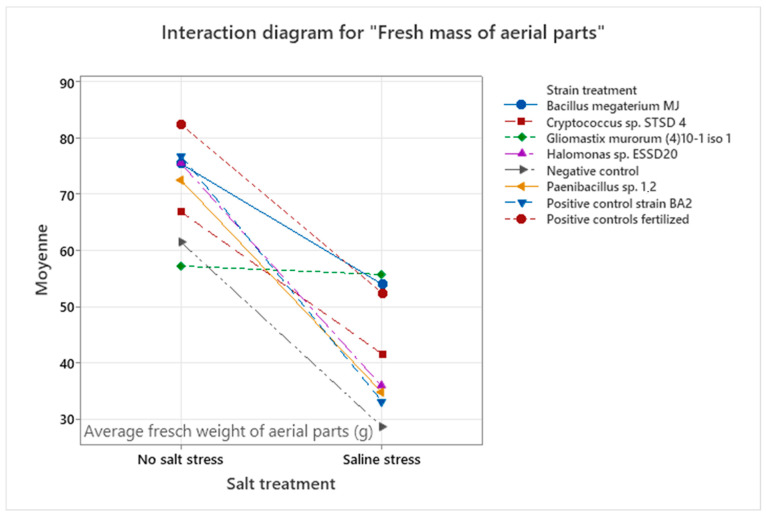
Two-factor ANOVA for the average fresh mass of the aerial parts of tomato plants showed a significant interaction between the two factors tested (*p*-value < 0.001).

**Figure A5:**
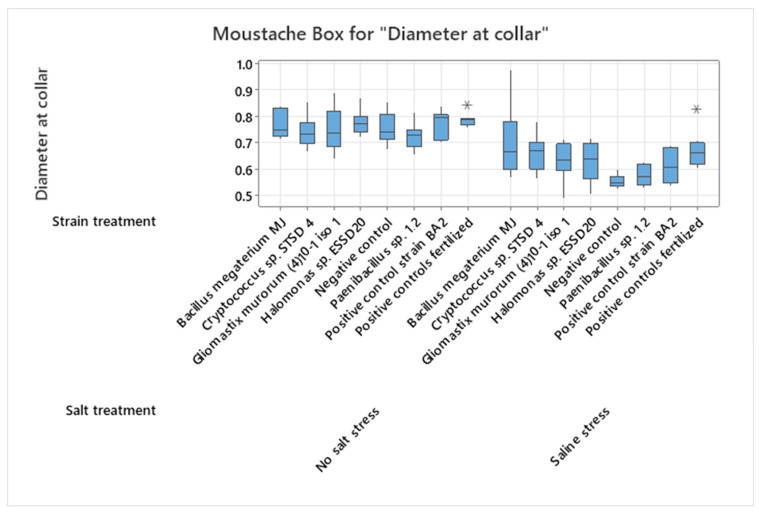
Mustache box diagram of neck diameters of tomato plants before subtraction of the 2 atypical values in the ‘salt stress’ group (in the fertilized positive control and the *B. megaterium* MJ strain treatments (* mark the presence of atypical values).

**Figure A6:**
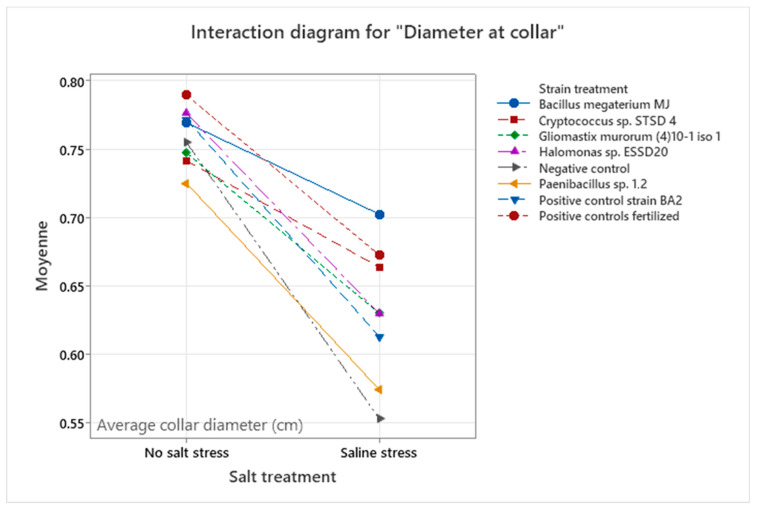
Interaction between the salt factor and the treatment factor. The interaction between the salt factor and the treatment factor was not significant (*p*-value = 0.130), but there was a weak interaction on the interaction diagram.

**Figure A7:**
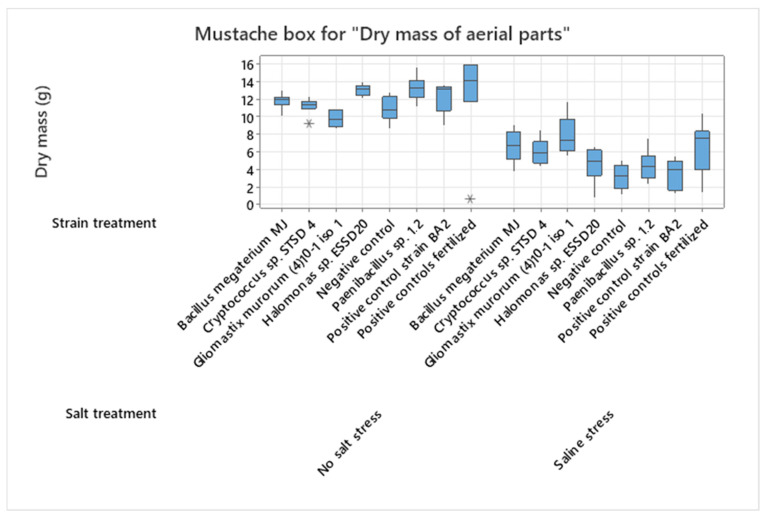
Mustache box diagram illustrating the distribution of data concerning the dry mass of the aerial parts of the in planta trial in the greenhouse after 6 weeks of cultivation (* mark the presence of atypical values).

**Figure A8:**
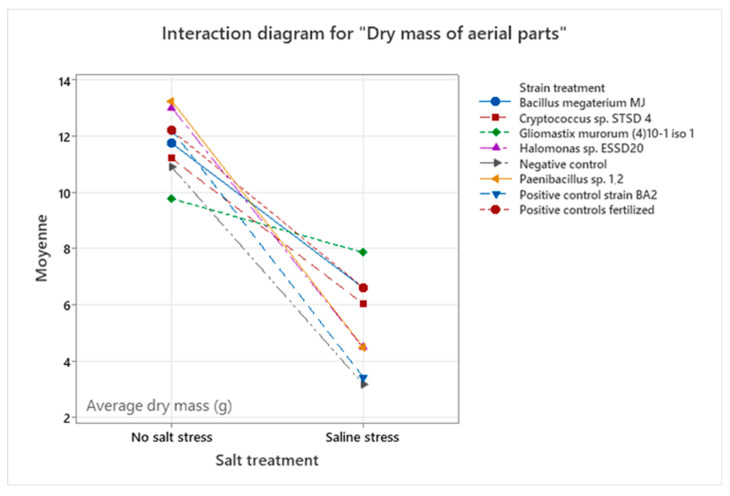
Diagram of interactions concerning the average dry mass harvested at the end of the in planta trial on tomato. The conditions for applying the two-factor ANOVA are not respected. However, this graph clearly illustrates the nature of the interaction between the two factors, which is significant (*p*-value < 0.0001).

**Figure A9:**
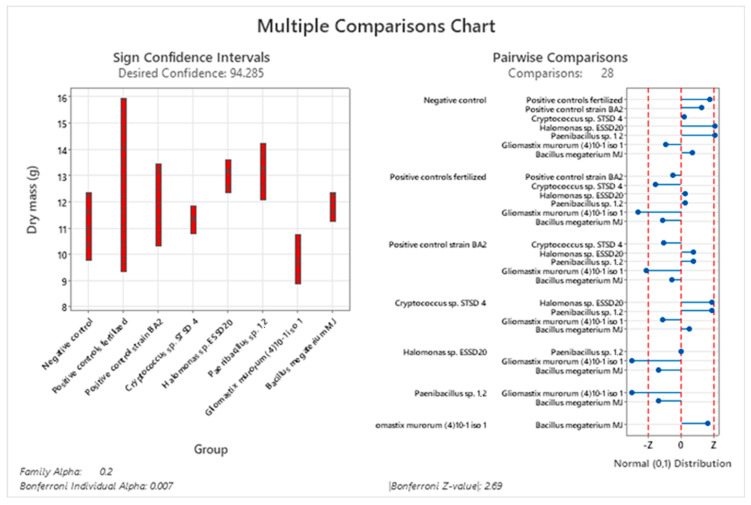
Kruskal–Wallis multiple comparison performed with the ‘Krus MC’ macro. This non-parametric test was used to compare the dry mass of the aerial parts as a function of the different treatments in the ‘no salt stress’ group of plants. This test compares the ranks of the observations.

**Figure A10:**
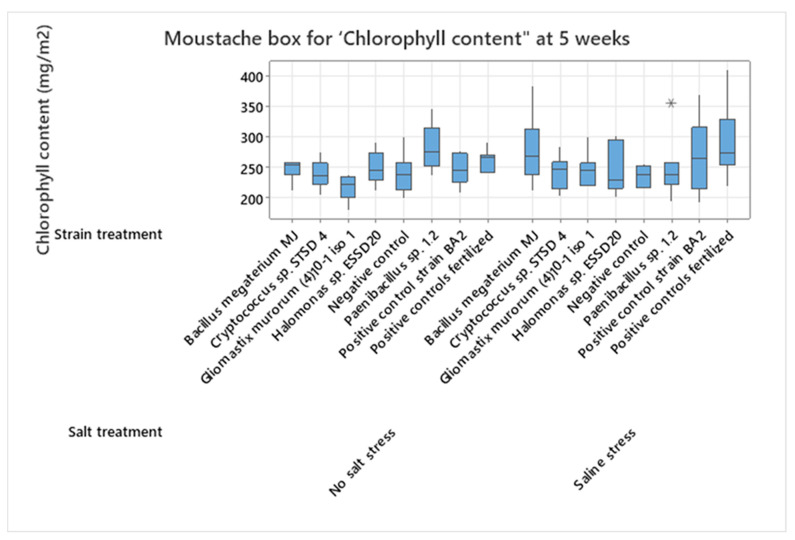
Mustache box diagram of ‘Chlorophyll content’ at 5 weeks, illustrating the distribution of data. It is clear that the variability is greater in the salt-treated group, which justifies the use of a non-parametric test for this group (* mark the presence of atypical values).

**Figure A11:**
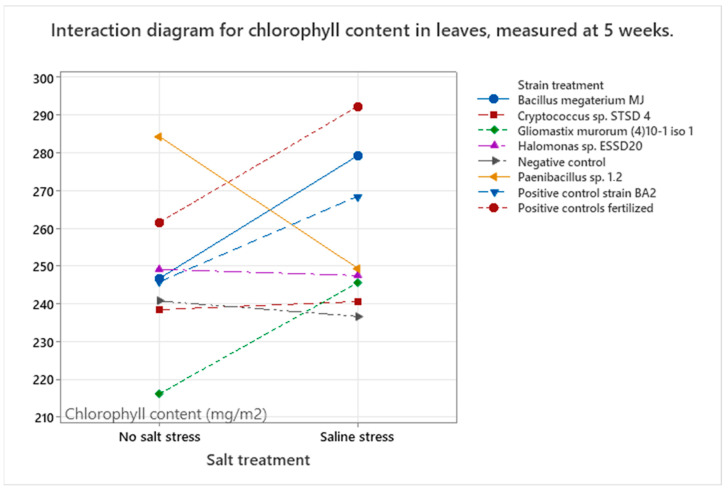
Diagram of interactions concerning the average chlorophyll content at 5 weeks. The conditions for applying the two-factor Anova are not respected. However, this graph clearly illustrates the nature of the interaction between the two factors, which is not significant (*p*-value = 0.138).

Some strains colonized the entire box, completely stopping the growth of the seedlings (Figure A1). This was observed for many bacteria of the *Bacillus* genus, in particular *Bacillus* sp. ESSD 22, *B. amyloliquefaciens* BA2, *B. amyloliquefaciens* CP, *Bacillus subtilis* 4.4, *Bacillus* sp. strain 44, *Bacillus* sp. 3.5, and *Bacillus* sp. E. The medium therefore needed to be modified to make it less rich by reducing the sucrose and yeast extract content, which was implemented in the second method.

In the second in vitro method, one of the two plates containing the *Cryptococcus* sp. STSD 4 strain under ‘salt stress’ conditions showed very poorly developed seedlings, in stark contrast to the other dish. Given that the seeds were selected randomly and homogeneously within the same batch, the hypothesis of climatic variations in the growth chamber seems the most plausible. According to the randomization plan, this box was placed against the back wall of the climate chamber, where temperatures were probably inappropriate for seedling development.

### 4.3. In Planta Test and Interpretation

The in planta test, conducted in greenhouse conditions, allowed for the evaluation of the impact of microorganisms on plant growth under both saline and non-saline conditions. The results from these trials showed a significant interaction between saline stress and the plant treatment.

Considering the fertilized positive control in the salt-stressed group, greater variability was observed between plants, though this treatment favored tomato growth. The addition of mineral salts to the pots, in addition to sodium chloride, had no deleterious effect. The fertilizer solution was added at a rate of 300 mL per pot after the salt solution had been applied. Although all the water (salt solution and fertilizer) remained in a closed circuit and collected in the cup, this water supplement probably had a diluting effect on the sodium chloride. Added to this is the effect of salt leaching towards the lower parts of the substrate and the cup, at least over a short period, which may have favored germination and seedling development. To limit this experimental bias, the fertilizer should be directly diluted in the salt solution in future experiments.

As the stress applied here is of a sodium nature, the addition of other ions, while increasing the total electrical conductivity of the solution, does not necessarily worsen the stress and may even have an antagonistic effect. This is particularly true of potassium, which plays a crucial role in mitigating salt stress [62,63,64,65]. The fertilizer used here contained potassium in the form of potassium oxide (K_2_O) at 74 g/L fertilizer.

*Cryptococcus* sp. STSD 4, under salt stress conditions, performed very well in the inoculation test on seedlings in vitro, ranking among the top two strains. However, this performance was not reproduced in the in planta greenhouse trial. The persistence of this yeast in the medium or its association with tomato plants could explain this difference. Optimization of the formulation could offer solutions to this problem, and further tests would be necessary to better understand the mechanisms involved.

The *Bacillus amyloliquefaciens* BA2 strain, already marketed as a biostimulant agent (Hélès), slightly enhanced the growth of tomato plants in the absence of salt stress, although this effect was not significant. This remains a simple trend, probably due to the low-limiting, low-stress conditions under which the strain was unable to express its full potential. On the other hand, under salt stress, this strain showed no growth-promoting activity. This is a tangible illustration that a strain effective under normal conditions is not necessarily so in the presence of abiotic stress. It is therefore essential to select strains adapted to the specific growth promotion objectives envisaged.

Out of the five selected strains, two showed no growth-promoting effect under salt stress: *Paenibacillus* sp. 1.2 and *Halomonas* sp. ESSD20. However, they did promote plant growth under non-stress conditions, notably by significantly increasing the dry biomass of above-ground parts. In addition, plants treated with *Paenibacillus* sp. 1.2 showed a higher chlorophyll content in their leaves.

Conversely, strains effective under salt stress conditions did not necessarily perform remarkably well in the absence of stress. For example, *Cryptococcus* sp. STSD 4 and *G. murorum* (4)10-1 iso 1 did not perform well in the no-salt conditions of the in planta trial. This strengthens the previous observations concerning the importance of adapting strain selection to the specific experimental conditions. However, it is interesting to note that both strains showed growth-promoting activity on seedlings in vitro, even in the absence of salt. One hypothesis is that in vitro seedlings evolve in a culture medium which, even without salt, is naturally unfavorable to their development, as shown by the growth difficulties of control seedlings, even in no-salt conditions.

*Bacillus megaterium* MJ appears to be more versatile, slightly enhancing the growth of tomato plants in the greenhouse test, although this effect was not significant under no-salt conditions.

The variations observed between fresh and dry biomass can be explained by differences in plant water content at the time of harvest. Despite efforts to maintain homogeneous humidity in the pots, fluctuations may persist, justifying the importance of dry biomass measurements for a more reliable assessment.

Finally, the NDVI values measured must be interpreted with caution. In fact, this method, mainly designed for outdoor cultivation, is less suited to isolated plants in pots. In this study, these measurements were taken for guidance only.

## 5. Conclusions

The results obtained from this study suggest that several tested microorganisms isolated from diverse sources (Table 1 and Table 5) have interesting potential for promoting tomato growth, particularly under saline stress conditions.

Strains such as *Halomonas* sp. ESSD20, *B. megaterium* MJ, *Cryptococcus* sp. STSD4, *G. murorum* (4)10-1 iso 1, and *Paenibacillus* sp. 1.2 demonstrated various abilities, ranging from stimulating seed germination to phosphate solubilization and IAA production during in vitro selection tests. The innovative in vitro seedling inoculation test has proved particularly effective in identifying strains with growth-promoting activities via various mechanisms in the presence of salt. This test provides a simple and practical method for sorting many strains, enabling them to be optimally adapted to the specific situation and plant species studied.

Three strains, namely, *B. megaterium* MJ, *Cryptococcus* sp. STSD4, and *G. murorum* (4)10-1 iso 1, stood out in the in planta greenhouse trial on tomatoes, notably boosting the biomass of aerial parts and the diameter of stems at the crown of plants exposed to a 200 mM saline solution at sowing and 400 mM during the trial, making them promising candidates for agricultural applications, especially in saline environments. These same strains showed no significant effect on tomato growth in the absence of salt stress. Conversely, the most effective strains in non-stressed conditions had no effect on salt-stressed plants. This underlines the importance of selecting strains specifically adapted to the targeted growth promotion context.

These microorganisms could potentially be used in strategies for sustainable management of saline soils, improving plant growth and reducing the need for chemical fertilizers. However, further studies, including larger-scale trials and analyses of the mechanisms behind the interactions between microorganisms and plants, are needed to validate these results and optimize their use under real-world cultivation conditions.

## Figures and Tables

**Table 1:** Selection of microorganism strains for in vitro screening tests from the P&P laboratory collection.

*Genus*	Species	Isolate Code	NCBI GenBank Accession	UASWS Code	Origin
*Bacillus*	*amyloliquefaciens*	BA2	KU060802.1	UASWS1212	Endophityc bacteria isolated from from *Aquilaria crassna*
*Bacillus*	*amyloliquefaciens*	CP	MT809121.1	UASWS2330	Bacteria isolated from curcuma powder
*Bacillus*	*aryabhattai*	5.3		UASWS3305	Bacteria isolated from decaying plane tree
*Bacillus*	*megaterium*	MJ	MG719638.1	UASWS1667	Endophytic bacteria isoled from Horse chestnut tree
*Bacillus*	*safensis*	B 23	MH671851.1	UASWS1806	Endophytic bacteria isoled from tomato stem
*Bacillus*	*subtilis*	4.4	MH500811.1	UASWS1742	Bacteria isolated from field pea seeds
*Bacillus*	sp.	strain 44	MT809118.1	UASWS2327	Endophytic bacteria isoled from Tomato stem
*Bacillus*	sp.	3.5	MH500806.1	UASWS1737	Bacteria isolated from bean seeds
*Bacillus*	sp.	BCb1	MT791119.1	UASWS2269	Bacteria isolated from *Malus domestica* leaves
*Bacillus*	sp.	E	ON103397	UASWS2601	Endophytic bacteria isoled from *Zantedeschia aethiopica*
*Cryptococcus*	sp.	STSD 4	MZ397906	UASWS2574	Endophytic yeast isoled from tomato seeds
*Gliomastix*	*murorum*	(4)10-1(iso1)	MW793376	UASWS2529	Fungus isolated from dune sand
*Kocuria*	*rhizophila*	B 3	MH671832.1	UASWS1787	Endophytic bacteria isoled from tomato stem
*Lysinibacillus*	sp.	SB 2	ON109155	UASWS2605	Endophytic bacteria isoled from tomato seeds
*Microbacterium*	sp.	P2R2	MN833674.1	UASWS2056	Bacteria isolated from decaying plane tree roots
*Paenibacillus*	*polymyxa*	BES 12			Bacteria isolated from sewage sludge
*Paenibacillus*	sp.	1.2	MH500810.1	UASWS1731	Bacteria isolated from wheat seeds
*Pantoea*	*agglomerans*	SB 6	ON109158	UASWS2608	Endophytic bacteria isoled from tomato seeds
*Pantoea*	*agglomerans*	P3R2	MN833675.1	UASWS2057	Bacteria isolated from decaying plane tree roots
*Pantoea*	sp.	PSb5	MT791118.1	UASWS2268	Bacteria isolated from *Malus domestica* leaves
*Pseudomonas*	sp.	SB 10	ON109161	UASWS2611	Endophytic bacteria isoled from tomato seeds
*Variovorax*	sp.	P1C1.4	MN833671.1	UASWS2053	Bacteria isolated from Malus domestica leaves

**Table 2:** PCR conditions for bacterial DNA amplification.

Primers	Steps	Temperature	Time	Cycles
27F/1492R	Initial denaturation	95 °C	1 min	
	Denaturation	95 °C	12 s	35 cycles
	Annealing	57 °C	12 s	
	Extension	72 °C	15 s	
	Final extension	72 °C	20 s	
	End of PCR	4 °C	∞	

**Table 3:** PCR conditions for fungal DNA amplification.

Primers	Steps	Temperature	Time	Cycles
ITS4/ITS5	Initial denaturation	95 °C	1 min	
	Denaturation	95 °C	12 s	32 cycles
	Annealing	55 °C	12 s	
	Extension	72 °C	10 s	
	Final extension	72 °C	20 s	
	End of PCR	4 °C	∞	

**Table 4:** In planta greenhouse trial. It included the 5 best performing strains from the selection tests, the strain *B. amyloliquefaciens* BA2 (from the commercial biostimulant Hélès), and a positive control with a chemical fertilizer. CFU concentrations of the inoculation solutions are given.

Species	Strain	Concentration of Inoculation Solution (CFU/mL)
*Halomonas* sp.	ESSD20	3.70 × 10^7^
*Bacillus megaterium*	MJ	6.20 × 10^4^
*Cryptococcus* sp.	STSD4	3.78 × 10^6^
*Gliomastix murorum*	(4)10-1 iso 1	4.43 × 10^5^
*Paenibacillus* sp.	1.2	2.90 × 10^7^
*Bacillus amyloliquefaciens*	BA2	5.20 × 10^5^
Other treatment		Concentration
*Commercial fertiliser*	Universal Fertilizer Wuxal G	20 g·L^−1^

**Table 5:** Genetic identifications of strains isolated from *S. europaea*.

Cultivation Method	Isolation Medium	Genus Species	Strain Code	NCBI GenBank Accession	UASWS Code
Disinfection, grinding and spreading	LBA	*Bacillus* sp.	ESAD 9	MZ397890.1	UASWS2579
		*Halomonas* sp.	ESAD 10	MZ397891.1	UASWS2580
		*Marinilactibacillus piezotolerans*	ESAD 11	MZ397892.1	UASWS2581
	YEA	*Thioclava* sp.	ESAD 12	MZ397893.1	UASWS2582
		*Marinilactibacillus piezotolerans*	ESAD 14	MZ397894.1	UASWS2583
	TSA	*Halomonas* sp.	ESAD 15	MZ397895.1	UASWS2584
		*Psychrobacter* sp.	ESAD 17	MZ397896.1	UASWS2585
Rinsing with water and cultivation of sections	LBA	*Psychrobacter* sp.	ESSD 19	MZ397897.1	UASWS2586
		*Halomonas* sp.	ESSD 20	MZ397898.1	UASWS2587
		*Pseudoalteromonas* sp.	ESSD 21	MZ397899.1	UASWS2588
	YEA	*Bacillus* sp.	ESSD 22	MZ397900.1	UASWS2589
		*Psychrobacter* sp.	ESSD 23	MZ397901.1	UASWS2590
	TSA	*Psychrobacter* sp.	ESSD 24	MZ397902.1	UASWS2591
		*Psychrobacter* sp.	ESSD 25	MZ397903.1	UASWS2592
		*Halomonas* sp.	ESSD 26	MZ397904.1	UASWS2593

**Table 6:** Average length of the main root and average number of secondary roots produced by tomato seedlings after inoculation of medium, in presence or absence of salt using method 1. Measures were carried out 14 days after staring the test.

Microorganism Strains	With Salt (50 mM NaCl)		Without (0 mM NaCl)	
	Average Length of Main Root (mm)	Average Number of Secondary Roots	Average Length of Main Root (mm)	Average Number of Secondary Roots
Negative control	23	1	19	2
*Bacillus* sp. *ESSD 22*	25	0	19	0
*Halomonas* sp. *ESSD 20*	32	2	20	1
*Psychrobacter* sp. *ESSD 23*	18	1	18	1
*Bacillus amyloliquefaciens BA2*	22	0	22	0
*Bacillus amyloliquefaciens CP*	21	0	18	0
*Bacillus aryabhattai 5.3*	19	2	16	2
*Bacillus megaterium MJ*	16	0	17	3
*Bacillus safensis B 23*	18	2	16	3
*Bacillus subtilis 4.4*	15	0	23	3
*Bacillus* sp. *strain 44*	16	0	26	2
*Bacillus* sp. *3.5*	17	1	23	1
*Bacillus* sp. *BCb1*	13	0	25	2
*Bacillus* sp. *E*	14	1	23	0
*Cryptococcus* sp. *STSD 4*	21	3	22	2
*Gliomastix murorum (4)10-1(iso1)*	18	2	18	1
*Kocuria rhizophila B 3*	16	2	24	2
*Lysinibacillus* sp. *SB 2*	22	0	22	1
*Microbacterium* sp. *P2R2*	19	1	20	0
*Paenibacillus polymyxa BES 12*	15	0	20	0
*Paenibacillus* sp. *1.2*	22	2	23	1
*Pantoea agglomerans SB 6*	16	2	20	0
*Pantoea agglomerans P3R2*	15	1	21	1
*Pantoea* sp. *PSb5*	20	1	25	1
*Pseudomonas* sp. *SB 10*	20	1	18	2
*Variovorax* sp. *P1C1.4*	18	2	16	2

**Table 7:** Germination rate, average length of the main root, average number of secondary roots, average stem length, and cotyledon emergence rate 21 days after the start of the test using method 2. This experiment only included the four best performing strains from the three selection tests along with a negative control. The germination rate represents the number of germinated seeds out of 10 seeds divided between two boxes. Non-germinated seeds and seedlings that fall out of the agar during germination are not considered for the other parameters evaluated (NA means no data available).

	Microorganism Strains	Germination Rate	Average Length of Main Root (mm) (±σ)		Average Number of Secondary Roots (±σ)		Average Stem Length (mm) (±σ)		Cotyledon Emergence Rate
With salt (50 mM NaCl)	Negative control	0%	NA		NA		NA		NA
	*Bacillus megaterium MJ*	0%	NA		NA		NA		NA
	*Cryptococcus* sp. *STSD 4*	100%	7	(±3.7)	3	(±3.7)	14	(±14.1)	56%
	*Gliomastix murorum (4)10-1(iso1)*	80%	16	(±5.3)	1	(±1.7)	20	(±6.1)	100%
	*Paenibacillus* sp. *1.2*	20%	2	(±0.7)	0	(±0)	0	(±0)	0%
Without (0 mM NaCl)	Negative control	60%	22	(±31.3)	0	(±0.8)	5	(±7.2)	17%
	*Bacillus megaterium MJ*	60%	17	(±6.9)	1	(±1.1)	13	(±10.3)	0%
	*Cryptococcus* sp. *STSD 4*	100%	19	(±12.6)	7	(±3.7)	22	(±8.6)	100%
	*Gliomastix murorum (4)10-1(iso1)*	90%	32	(±2.7)	10	(±2.3)	32	(±6.4)	100%
	*Paenibacillus* sp. *1.2*	40%	4	(±4)	0	(±0)	0	(±0)	0%

**Table 8:** Treatments statically different from the negative control of the respective group according to the different parameters analyzed. Confidence indices are shown where possible (parametric test).

Modality		Fresh Mass of Aerial Parts (g) [Simultaneous Bonferroni 95% Confidence Interval]	Diameter at the Stem Neck of Tomato Plants (cm) [Simultaneous Dunnet 95% Confidence Interval]	Dry Weight of Aerial Parts of Tomato Plants (g) [Simultaneous Dunnet 95% Confidence Interval]	Chlorophyll Content in Leaves (mg/m^2^) [Simultaneous Dunnet 95% Confidence Interval]
**Salt stress**	**(*Bacillus megaterium* MJ) − (Negative control)**	[8.89; 41.80]	[0.018; 0.204]	[0.62; 6.23]	-
	**(*Cryptococcus* sp. STSD 4) − (Negative control)**	-	[0.021; 0.201]	[0.05; 5.66]	-
	**(*Gliomastix murorum* (4)10-1 iso 1) −** **(Negative control)**	[10.62; 43.53]	-	[1.88; 7.48]	-
	**(Positive controls fertilized) − (Negative control)**	[7.33; 40.24]	[0.005; 0.190]	[0.62; 6.22]	-
**No salt stress**	**(*Paenibacillus* sp. 1.2) − (Negative control)**	-	-	-	[8.72; 78.28]

**Table 9:** Average diameter of the stem neck, dry weight of aerial parts, average chlorophyll content, and NDVI. The measurements of stem diameter at the collar and chlorophyll content of the leaves were carried out five weeks after the start of the experiment.

Salt Treatment	Strain Treatment	Average of Diameter at the Stem Neck of Tomato Plants ^(1)(1’)^ (cm) (±σ)		Dry Weight of Aerial Parts of Tomato Plants					Average of Chlorophyll Content in Leaves ^(4)(4’)^ (mg/m^2^) (±σ)		NDV Index ^(5)^
				Average ^(2)^ (g)	(±σ)	Average (g)	(±σ)	Median ^(3)^			
Salt stress	Negative control	0.553	(±0.025)	3.18 ^A^	(±1.46)				237	(±17)	0.35
	*Halomonas* sp. *ESSD20*	0.630	(±0.074)	4.48 ^AB^	(±1.91)				248	(±40)	0.37
	*Bacillus megaterium MJ*	0.664 (*)	(±0.085)	6.61 ^BC^	(±1.77)				279	(±54)	0.57
	*Cryptococcus* sp. *STSD 4*	0.664 (*)	(±0.068)	6.04 ^ABC^	(±1.41)				241	(±27)	0.34
	*Gliomastix murorum (4)10-1 iso 1*	0.630	(±0.073)	7.86 ^C^	(±2.13)				246	(±27)	0.56
	*Paenibacillus* sp. *1.2*	0.575	(±0.038)	4.50 ^AB^	(±1.61)				249	(±47)	0.43
	Positive control strain BA2	0.613	(±0.063)	3.44 ^A^	(±1.70)				269	(±61)	0.44
	Positive controls fertilized	0.651 (*)	(±0.037)	6.60 ^BC^	(±2.98)				292	(±60)	0.5
No salt stress	Negative control	0.755	(±0.061)			10.90	(±1.43)	10.75	241 ^AB^	(±32)	0.47
	*Halomonas* sp. *ESSD20*	0.776	(±0.046)			13.01	(±0.66)	13.12 (*)	249 ^ABC^	(±26)	0.56
	*Bacillus megaterium MJ*	0.770	(±0.053)			11.75	(±0.88)	11.95	247 ^ABC^	(±16)	0.67
	*Cryptococcus* sp. *STSD 4*	0.742	(±0.059)			11.21	(±0.89)	11.38	239 ^AB^	(±22)	0.58
	*Gliomastix murorum (4)10-1 iso 1*	0.748	(±0.084)			9.77	(±0.87)	9.66	216 ^A^	(±21)	0.48
	*Paenibacillus* sp. *1.2*	0.725	(±0.050)			13.26	(±1.40)	13.25 (*)	284 ^C^	(±37)	0.52
	Positive control strain BA2	0.772	(±0.053)			12.23	(±1.73)	13.18	246 ^ABC^	(±25)	0.56
	Positive controls fertilized	0.790	(±0.027)			12.21	(±5.41)	14.05	262 ^BC^	(±18)	0.58
	**(1)** Salt stress group only: One-factor anova: treatment effect (*p*-value = 0.011). Dunnett’s test at 95% confidence level: treatments marked with (*) are statistically different from the negative control. These are the results recorded 5 weeks after the launch of the trial.										
	**(1′)** No salt stress: Non-parametric Kruskal–Wallis test: no effect of the ‘strain’ treatment (*p*-value unadjusted = 0.291).										
	**(2)** One-factor anova: Effect of the ‘strain’ treatment (*p*-value < 0.001). Groupings made using the Tukey method at a 95% confidence level (different letters show statistical significance).										
	**(3)** Non-parametric Kruskal–Wallis test: Effect of the ‘strain’ treatment (*p*-value < 0.001). Multiple comparison using the ‘Kru MC’ macro. Treatments marked with (*) are statistically different from the negative control.										
	**(4)** Salt stress: non-parametric Kruskal–Wallis test: No effect of the ‘strain’ treatment (*p*-value unadjusted = 0.302).										
	**(4′)** No salt stress: One-factor anova:effect of the ‘strain’ treatment (*p*-value < 0.001). Groupings made using the Tukey method at a 95% confidence level (different letters show statistical significance).										
	**(5)** Normalized Difference Vegetation Index: Average of three measurements taken on all plants in each modality.										
	***General remarks****:* At least one seed germinated in each of the 128 pots. Four plants died during the trial and are therefore not taken into account in the statistical analysis. Two plants died in the ‘negative control with salt stress’, one in the ‘positive control fertilised without salt stress’ and one in the ‘positive control BA2 strain without salt stress’.										

## Data Availability

All DNA sequences obtained or mentioned in this work are available at https://www.ncbi.nlm.nih.gov/nucleotide/. GenBank accessions are available in Table 1.
